# Persistent Unexplained Dyspnea: A Case of Hepatopulmonary Syndrome

**DOI:** 10.1155/2017/1469893

**Published:** 2017-08-29

**Authors:** Alfonso Campanile, Alessandro Colombo, Maurizio Del Pinto, Claudio Cavallini

**Affiliations:** Department of Cardiology, Hospital “S. M. della Misericordia”, Perugia, Italy

## Abstract

Regarding a patient with dyspnea, the history and physical examination often lead to the correct diagnosis. In some circumstances, when more than one underlying disease is present, the diagnostic process can be more challenging. We describe an unusual case of dyspnea and persistent hypoxemia related to a hepatopulmonary syndrome in a 53-year-old patient with known heart failure and chronic liver disease. Initially managed with intravenous diuretic therapy, due to signs of lung and peripheral congestion, our patient did not improve as expected; therefore we performed more advanced studies with a chest-abdomen CT scan and a right heart catheterization. They showed, respectively, no signs of parenchymal and vasculature lung disease, a cirrhotic liver disease, splenomegaly, signs of portal hypertension, and high cardiac output with normal pulmonary vascular resistance. These results, along with the association of hypoxemia and chronic liver disease, suggested a hepatopulmonary syndrome. The diagnosis was confirmed by the demonstration of an intrapulmonary vascular dilatation with right to left shunt during a microbubble transthoracic echocardiography and a lung perfusion scan. Liver transplantation is the only successful treatment for this syndrome; however, the patient became soon unsuitable for this strategy, due to a rapid clinical deterioration.

## 1. Introduction

Dyspnea is a common symptom that affects up to 50% of patients admitted to acute, tertiary care hospitals and a quarter of patients seeking care in ambulatory settings [1]. In specialty practice, patients with chronic dyspnea account for 15–50% of those seen by cardiologists and just under 60% of those seen by pneumonologists [2]. The distribution of underlying diagnoses varies from one care situation to another; however, lung and heart pathologies represent the most frequent causes [3]. In 30–50% of cases, particularly in elderly and multimorbid patients, the diagnosis can be challenging and more information is needed [3].

We present herein a case of dyspnea in a 53-year-old man with several morbidities, where the symptom was initially interpreted as a manifestation of heart failure but, after extensive investigations, related to a more rare condition: the hepatopulmonary syndrome (HPS).

## 2. Case Presentation

**Figure 1:**
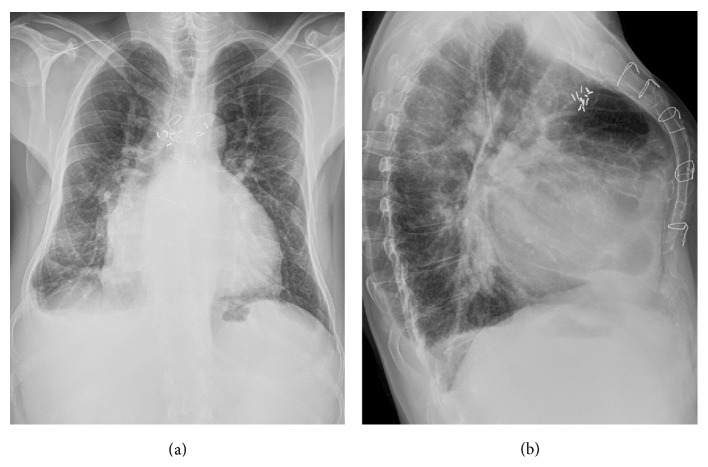
Two projection chest X-rays with the anteroposterior view on the left and the lateral view on the right, showing signs of pulmonary congestion and a right basal pleural effusion.

**Figure 2:**
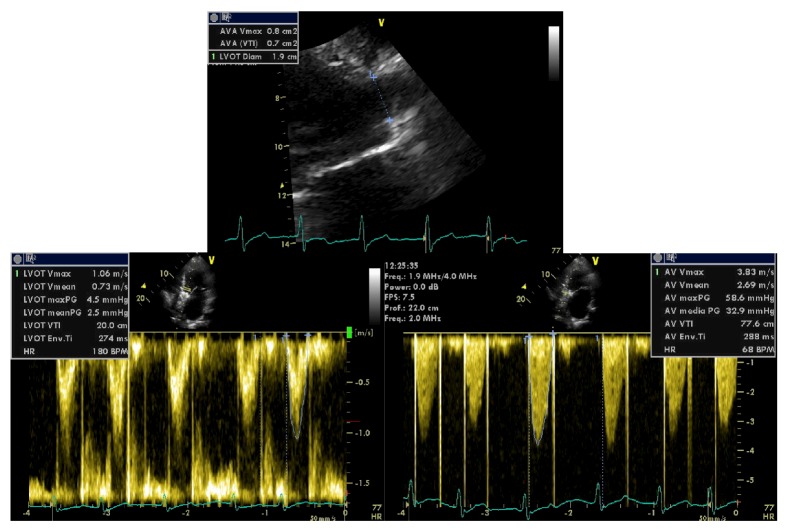
Effective orifice area calculation for the mechanical prosthesis on the transthoracic echocardiography. On the top, the left ventricle outflow tract (LVOT) diameter measurement; on the bottom, the pulse wave analysis on the LVOT on the left and the prosthesis continuous wave analysis on the right.

**Figure 3:**
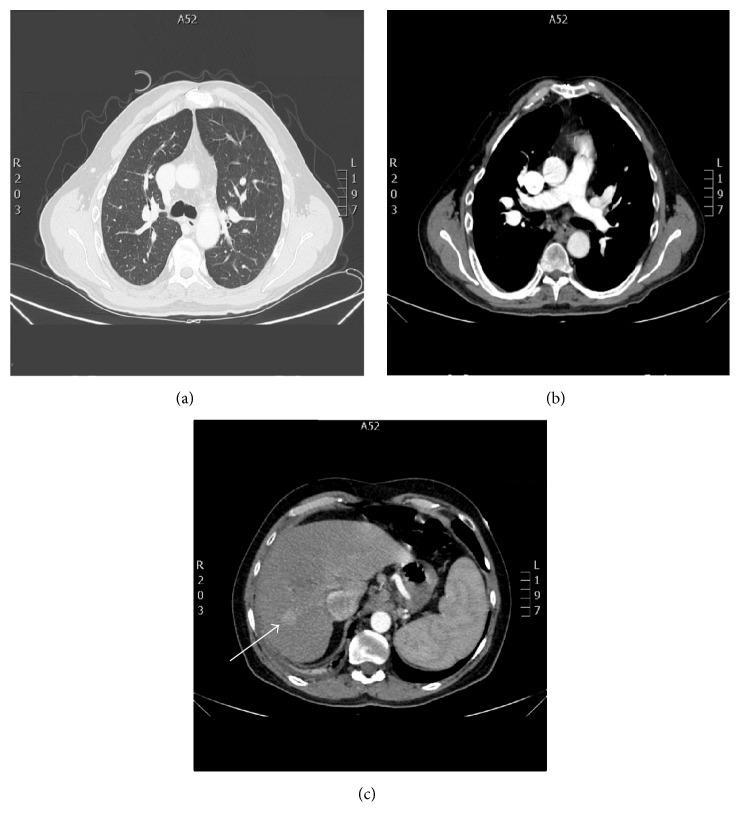
Parenchymal (a) and pulmonary vasculature study (b) on the chest CT scan showing no pathological findings; (c) abdomen CT scan showing a cirrhotic liver with a focal lesion (white arrow), highly suspicious of hepatocellular carcinoma.

**Figure 4:**
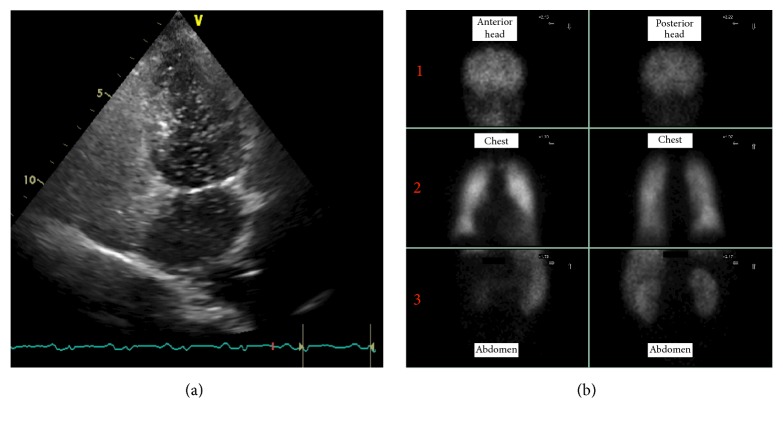
(a) Microbubble transthoracic echocardiography showing a right to left shunt with a delayed opacification (>3 cardiac cycles) of the left atrium and ventricle; (b) macroaggregated albumin lung perfusion scan (MAA scan) showing radioactivity in the lungs (panel 2) as well as in the cerebrum (panel 1) and in the kidneys (panel 3), suggesting right to left shunt.

A 53-year-old man was admitted to our cardiology department due to a history of progressive dyspnea (NYHA class III). His medical background was characterized by a congenital heart disease (interventricular septal defect, surgically corrected when he was 10 with a dacron patch apposition), aortic valve and interventricular patch replacement (mechanical St. Jude 25 and a new dacron patch), respectively, for severe aortic regurgitation and interventricular defect recurrence (Laubry-Pezzi syndrome), permanent atrial fibrillation in oral anticoagulant therapy, chronic liver disease (HCV-related), monoclonal gammopathy of undetermined significance (MGUS), pancytopenia, severe mitral and tricuspid regurgitation with pulmonary hypertension, and a previous admission with a diagnosis of heart failure with preserved ejection fraction (HFpEF). The main clinical and laboratory parameters are summarized in Table 1. The physical examination revealed jugular venous distension, an irregular cardiac rhythm with a 2/6 systolic murmur over the aortic and mitral valve, a significant reduction of breath sounds on the right basal zone, and a peripheral edema. An atrial fibrillation with a left bundle branch block (LBBB) was detected on the electrocardiogram (ECG) and a chest X-ray revealed signs of pulmonary congestion and a right basal pleural effusion (Figure 1). The transthoracic echocardiogram (TTE) showed a dilated left ventricle with preserved EF (60%), a dilated right ventricle with normal systolic function, no residual shunt at the interventricular septum, aortic mechanical prosthesis in a correct position, with preserved discs opening, no signs of pathological intra- or periprosthesis regurgitation, increased transprosthetic gradients (mean gradient of 33 mmHg), and an effective orifice area index (EOAi) of 0,4 cm^2^/m^2^ suggesting severe prosthesis-patient mismatch (PPM) (Figure 2) [4, 5]. Moderate mitral and severe tricuspid regurgitation were documented as well, with an estimated systolic pulmonary pressure (sPAP) of 50 mmHg. No pericardial effusion was detected and a dilated inferior vena cava with poor collapsibility during inspiration was evident. Both clinical and instrumental data suggested a diagnosis of HFpEF and an intravenous diuretic treatment was started. Despite good response to the therapy, documented both clinically (reduction of peripheral edema) and radiographically (a second chest X-ray did not document signs of pulmonary congestion), the patient remained symptomatic for dyspnea with low exercise tolerance. The oxygen therapy was still needed to maintain a satisfactory blood oxygenation. We decided, therefore, first to study the pulmonary parenchyma and vasculature system with a contrast CT scan and second to perform, guided by the CT results, a right heart catheterization. A chest-abdomen CT examination was carried on with evidence of small right pleural effusion, no signs of lung parenchymal disease and pulmonary embolism (Figures 3(a) and 3(b)), cirrhotic liver disease with a focal lesion highly suspicious for hepatocellular carcinoma, splenomegaly, and signs of portal hypertension (Figure 3(c)). The hemodynamic study was then scheduled and revealed high cardiac output and normal pulmonary vascular resistance with only a slight increase of the mean pulmonary pressure and wedge pressure. The intravenous diuretic therapy was carried on; however the high cardiac output state and the results of the abdominal CT scan pushed us towards other hypothesis. A gastroenterology consultant review was requested and the hypothesis of a hepatopulmonary syndrome was advanced. A microbubble TTE (MTTE) and a lung scintigraphy (99 mTc-MAA) were performed with evidence of intrapulmonary right to left shunt (Figures 4(a) and 4(b)). The diagnosis of HPS was confirmed and the patient was transferred to the gastroenterology department for the specific treatment required. In less than three months, his medical condition rapidly deteriorated despite treatment. He developed multifocal hepatocellular carcinoma, liver failure, and severe encephalopathy. Due to rapid evolution of the clinical picture, he was unsuitable for transplantation and moved under the management of the palliative care team.

## 3. Discussion 

The HPS is a severe and common pulmonary vascular complication of liver disease with a prevalence varying from 4 to 47% [6]. The diagnosis, based on the classic triad of hepatic disease, arterial deoxygenation, and intrapulmonary vascular dilatation (IPVD) [7], can often be missed due to nonspecific presentation and presence of other comorbidities [8]. Our case well illustrates this challenging situation. The most evident symptom of HPS is dyspnea; however, other clinical manifestations, more commonly seen in this syndrome, despite not specific for it, are platypnea (increased shortness of breath while sitting up from prone position), orthodeoxia (decrease in arterial saturation by more than 5% or 4 mmHg in erect position), spider nevi, and clubbing [9]. Apart from progressive dyspnea, none of these features were present in our patient during the admission, which contributed to the delay in our diagnosis. Another peculiar characteristic of our case is that the severity of liver disease was accidentally brought to light by investigations performed with a different aim: the definition of the dyspnea causes. A similar pattern was described in a previous case report, where the HPS guided the diagnosis of cirrhosis [10]; however, in that case, the patient did not have any documented history of liver disease while our patient presented to our attention with a history of liver disease related to HCV infection. In relation to this aspect it is important to point out that there is no direct correlation between severity of HPS and the underlying liver disease [11]. Since his admission the patient showed two of the three main characteristics of the HPS: liver disease and low pulse oximetry saturation. To make a definite diagnosis we needed to demonstrate an IPVD with a right to left shunt. We decided, therefore, to perform both a MTTE and a 99 mTc-MAA. The first is, indeed, considered the gold standard for diagnosing intrapulmonary shunting [12, 13]. The second one does not distinguish intracardiac and intrapulmonary shunting and has inferior sensitivity compared to MTTE; however, it may help to distinguish the degree of hypoxemia caused by IPVD (abnormal brain uptake > 6%) in the setting of coexistent intrinsic cardiopulmonary disease [13]. Specifically, our patient showed a brain uptake equal to 19%, suggesting a predominant contribution of the right to left shunt in the severity of his hypoxemia. Currently, no effective medical therapies exist for the hepatopulmonary syndrome, and liver transplantation is the only successful treatment, with improvement or complete resolution in hypoxia in 85% of cases within 1 year [10]. Supplemental oxygen to maintain oxygen saturation > 88% is frequently recommended, but there are no data to confirm clinical benefits [14]. The mortality is significantly worse (doubled) in cirrhotic patients with HPS in comparison to patients without HPS; that is why patients with HPS may be eligible to receive a higher priority on the transplant waitlist, depending on the allocation system [13, 15]. HPS is usually asymptomatic, so all liver transplant centers should routinely screen patients for HPS [6]. In our case the combination of different factors (severe cardiac disease and unrecognized advanced liver disease) contributed to a delayed diagnosis with subsequent limited therapeutic options which resulted in the poor outcome outlined.

## 4. Conclusion

The hepatopulmonary syndrome is a severe complication of liver disease and it significantly affects the patient's prognosis. Its diagnosis is complicated because it is often asymptomatic and when it becomes evident, its clinical manifestations (e.g., dyspnea) are not specific and can easily be related to other more frequent pathological conditions. We should always consider this condition in the differential diagnosis of unexplained hypoxemia in a patient affected by hepatic disease.

## Figures and Tables

**Table 1:** Main clinical and laboratory parameters.

*Clinical parameters*	
Temperature (°C)	36.4
Blood pressure (mmHg)	110/45
Mean heart rate (bpm)	85
Peripheral oxygen saturation in room air (%)	82
Partial pressure of oxygen in room air (mmHg)	42
Peripheral oxygen saturation (%) during oxygen treatment with a fraction of inspired oxygen (FiO2) of 60%, *supine position*	97
Peripheral oxygen saturation (%) during oxygen treatment with a fraction of inspired oxygen (FiO2) of 60%, *sitting position*	95

*Laboratory findings *	
Haemoglobin (g/dl)	10,6
Hematocrit (%)	31,7
Mean corpuscular volume (MCV, fl)	107,5
Platelet count (×10^3^/*μ*L)	49
White cell count (×10^3^/*μ*L)	2,95
International normalized ratio (INR)	3,34
Creatinine (mg/dl)	0,79
Azotemia (mg/dl)	34
Potassium (mEq/L)	4,0
Total bilirubin (mg/dl)	1,74
Conjugate bilirubin level (mg/dl)	1,17
Aspartate aminotransferase (AST/GOT, IU/L)	73
Alanine aminotransferase (ALT/GPT, IU/L)	31
